# 6-Bromo-2-naphthol from *Silene armeria* extract sensitizes *Acinetobacter baumannii* strains to polymyxin

**DOI:** 10.1038/s41598-022-11995-y

**Published:** 2022-05-20

**Authors:** Mingyeong Kang, Wonjae Kim, Jaebok Lee, Hye Su Jung, Che Ok Jeon, Woojun Park

**Affiliations:** 1 Laboratory of Molecular Environmental Microbiology, Department of Environmental Science and Ecological Engineering, Korea University, Seoul, 02841 Republic of Korea; 2 Department of Life Science, Chung-Ang University, Seoul, 06974 Republic of Korea

**Keywords:** Antimicrobials, Applied microbiology

## Abstract

The overuse of antibiotics has led to the emergence of multidrug-resistant bacteria, which are resistant to various antibiotics. Combination therapies using natural compounds with antibiotics have been found to have synergistic effects against several pathogens. Synergistic natural compounds can potentiate the effects of polymyxins for the treatment of *Acinetobacter baumannii* infection. Out of 120 types of plant extracts, only *Silene armeria* extract (SAE) showed a synergistic effect with polymyxin B (PMB) in our fractional inhibitory concentration and time-kill analyses. The survival rate of *G. mellonella* infected with *A. baumannii* ATCC 17978 increased following the synergistic treatment. Interestingly, the addition of osmolytes, such as trehalose, canceled the synergistic effect of SAE with PMB; however, the underlying mechanism remains unclear. Quadrupole time-of-flight liquid chromatography-mass spectrometry revealed 6-bromo-2-naphthol (6B2N) to be a major active compound that exhibited synergistic effects with PMB*.* Pretreatment with 6B2N made *A. baumannii* cells more susceptible to PMB exposure in a time- and concentration-dependent manner, indicating that 6B2N exhibits consequential synergistic action with PMB. Moreover, the exposure of 6B2N-treated cells to PMB led to higher membrane leakage and permeability. The present findings provide a promising approach for utilizing plant extracts as adjuvants to reduce the toxicity of PMB in *A. baumannii* infection.

## Introduction

*Acinetobacter baumannii* is a medically important pathogen having high resistance to major antibiotics (e.g., carbapenems and ciprofloxacin) but low resistance to polymyxin B (PMB)^1^. For the control of MDR bacteria, including *A. baumannii*, new antibiotics and strategies need to be explored. The discovery of new antibiotics to combat MDR bacteria can be possible if a new antibiotic-producing bacterium is discovered from natural environments^2^. However, this approach has disadvantages in that it is time-consuming, and newly developed chemicals may be toxic to humans. To overcome these shortcomings, methods of mixing antibiotics with/without natural chemicals have often been found to exhibit synergistic effects against several MDR bacteria^3^. Reactive oxygen species-generating oleanolic acid (OA) from natural plants has been proven to exhibit synergism with aminoglycoside antibiotics to treat *A. baumannii* infection^4^.

Natural antimicrobial components are known to have lower side effects and higher therapeutic potentials than artificially synthesized antimicrobials. There are more than 250,000 to 500,000 types of plants on the earth, and many plant-derived antimicrobial agents have been widely used in medical fields^5^. *Salvadora persica* L. extract has antimicrobial activity against several pathogens, including *Streptococcus faecalis* and *Candida albicans*^6^. Moreover, grape pomace extract has been found to exhibit synergistic effects with β-lactam, quinolone, fluoroquinolone, tetracycline, and amphenicol against *S. aureus* and *E. coli* strains by inducing cytoplasmic membrane damage^7^. Many plant extracts possess heterogeneous groups of phytochemicals that are often bioactive phenolic compounds, e.g., eugenol from clove oil, thymol from *Thymus vulgaris*, and carvacrol from oregano. These biologically effective, hydroxylated phenolic molecules can disintegrate bacterial membranes because of their low molecular weights and slightly high water solubility^8^. Several other plant-derived secondary metabolites, such as alkaloids, flavonoids, tannins, and quinone compounds, have antimicrobial properties. Their combination with antibiotics could lower the concentration of antibiotics and sensitize MDR pathogens to antibiotics. For example, oregano, essential oil from *Origanum vulgare*, has been found to have a synergistic effect with PMB against MDR *A. baumannii* strains possessing both metallo-β-lactamase (MβL) and carbapenemase by disrupting the bacterial cell membrane^9^.

Polymyxins are cationic amphipathic compounds that mainly kill gram-negative bacteria by selectively acting on lipid A of lipopolysaccharide (LPS), leading to increased membrane permeability and damaged cellular membranes. PMB-exposed cells are known to modify the phosphate of lipid A with phosphoethanolamine (PetN) or positively charged 4-amino-4-deoxy-l-arabinose, thereby increasing the bacterial surface charge, and reducing the binding of PMB to outer membranes^10^. In PMB-resistant *A. baumannii*, the two-component system PmrAB-induced *pmrCAB* is responsible for increased resistance to PMB by the addition of PetN to a PetN transferase encoded by the *pmrC* gene^11,12^. Although it has considerable nephrotoxicity and neurotoxicity in humans, PMB is still used as the last resort to treat bacterial infections. Combination therapies with natural compounds may be desirable to reduce the amounts of PMB possibly used for controlling MDR pathogen^13^. In our study, the extracts obtained from *Silene armeria*, a plant species belonging to the family Caryophyllaceae were found to have a synergistic effect with PMB against *A. baumannii*. 6-Bromo-2-naphthol (6B2N) was identified as an effective compound to reduce the minimum inhibitory concentration (MIC) of PMB for killing *A. baumannii*. Our findings provide new directions for the effective control of MDR *A. baumannii* using a combination of PMB and natural extracts.

## Materials and methods

### Bacterial strains, clinical isolates, and culture conditions

The bacterial strains used in the present study are listed in Table S5. In total, 15 clinical isolates and five different genera (*Acinetobacter, Escherichia, Pseudomonas, Salmonella*, and *Listeria*) were investigated. The clinical *A. baumannii* isolates were obtained from patients treated at the National Culture Collection for Pathogens (NCCP). All the isolates were grown at 37 °C in Luria–Bertani (LB) broth with aeration by shaking at 220 rpm overnight. The overnight cell cultures were diluted 1/100 in fresh LB broth and incubated at 37 °C for 3 h. The growth of cultured cells was determined by measuring the optical density at 600 nm (OD_600_).

### Determination of PMB susceptibility using plant extracts

To determine the MIC of antibiotics, the following five antibiotics (Sigma, USA) were used in the present study: PMB, meropenem, doxycycline, gentamicin, and erythromycin^14^. The natural extracts were provided by the Freshwater Bioresources Culture Collection (FBCC) of the Nakdonggang National Institute of Biological Resources. Their extractions were performed using 70% ethanol, methanol, or water, which was described at the FBCC (https://fbp.nnibr.re.kr/fbcc) in detail. In total, 120 types of natural extracts were used in the experiments (Table S1). The extracts from the FBCC were dissolved in dimethyl sulfoxide (DMSO, 20 mg/mL) and various concentrations (32–512 µg/mL) were prepared to screen synergistic effects with PMB. All the natural extracts were stored at − 20 °C. The same volume of dimethyl sulfoxide was used as the negative control. The microdilution checkerboard method was used to assess the synergistic effect of antibiotic treatment alone and combination treatment with the antibiotic and natural extracts in each 96-well plate. Cell growth was measured at 37 °C for 24 h using a microplate spectrophotometer (Spark, Germany). FIC index (FICI) was calculated using the following equation: (MIC of compound A in combination)/(MIC of compound A alone) + (MIC of compound B in combination)/(MIC of compound B alone). FICI ≤ 0.5 indicates synergy, FICI > 0.5 but ≤ 4 indicates no interaction, and FICI > 4 indicates antagonism. After the screening step, *Silene armeria,* a garden plant available in any agricultural market in the Republic of Korea was purchased to test our further experiments for the identification of active compounds. *Silene armeria* extracts obtained by ethanol (70%) extraction (hereinafter referred to as SAE) were dried, weighed, and used in all experiments after preparing dilutions from stock solutions (20 mg/mL in DMSO), except for the Q-TOF–LC/MS analysis. All experimental procedures using plant materials including the collection of the FBCC plant extracts complied with the guidelines and legislation of our institution, funding agencies, and national standards.

### Time-kill assay

The time-kill assay of *A. baumannii* ATCC 17978 was performed with some modifications^4^. Exponentially grown cells were diluted (1/100) with LB liquid medium (5 mL) and the final 10^7^ CFU/mL cells were used for the time-kill assay. Then, the cells were treated with PMB (4 µg/mL) and SAE (128 µg/mL) in autoclaved LB broth (10 mL). Cells were harvested at each time point (0, 1, 2, 3, 4, 5, 15, 30, 45, and 60 min) and washed twice with PBS, following which 100 μL aliquots were transferred to LB agar plates for colony counting. The plates were incubated at 37 °C overnight.

### SEM and TEM

SEM and TEM were conducted to visualize the synergistic effect of *A. baumannii* cells. Overnight cultured *A. baumannii* cells were diluted (1/100) and then were grown in fresh LB media to make the final tested cell density (10^6^ CFU/mL). Then, *A. baumanii* cells were treated with PMB (2 μg/mL) and SAE (256 μg/mL) for 15 min per sample. The samples were fixed at 4 °C for 12 h (Karnovsky’s fixative) and washed twice with cold (4 °C) potassium phosphate buffer (0.05 M) for 10 min each. The step of fixation was processed with a mixture of potassium phosphate buffer (0.1 M) and OsO_4_ (2%) at 4 °C for 2 h and washed twice with double-distilled water (ddH_2_O) at room temperature. The cells were dehydrated using increasing ethanol concentrations [30, 50, 70, 80, and 90%, and three times with ethanol (100%)] for 10 min each at room temperature^15^. The samples were coated with platinum before examination using FE-SEM (FEI, Japan). TEM was performed at the Korea University Medical Research Center (https://medicine.korea.ac.kr/web/msrc/home). Aliquots were transferred onto carbon-coated copper grids and fixed with 2.5% glutaraldehyde diluted in 50 mM sodium cacodylate (pH 7.2). After washing thrice with 3% saccharose, the cells were observed. For bacterial cell imaging, aliquots of purified samples were dispensed onto carbon-coated copper grids. Excess liquid was discarded from the grids, and the samples were negatively stained with phosphotungstic acid (3% [wt/vol]) for 5 min and dried using filter paper. The specimens were examined using an H-7100 microscope (Hitachi, Tokyo, Japan) operating at an accelerating voltage of 75 kV and a magnification of × 40,000 for cells.

### Survival rate by pretreatment exposure time to plant extracts

The SAE (128 μg/mL) was placed in autoclaved LB medium (10 mL). Then, pretreatment of exponentially grown *A. baumannii* cells with SAE was conducted by inoculating cells (10^6^ CFU/mL) into the same test tube. Cells were harvested at each time point (0, 30, 60, and 120 min), and washed twice with PBS. The pretreated cells were subsequently treated with PMB (1 μg/mL) in LB medium (10 mL) for 30 min. In total, 1 mL of treated cells were collected and washed twice with PBS. The number of survived cells was measured by serially diluting cells with LB media and spotting each dilution on LB agar plates to obtain their survival rate. The plates were incubated at 37 °C overnight.

### Measurement of zeta potential and cell integrity

The surface zeta potential of *A. baumannii* cells was measured at 25 °C in an ELSZ-1000 Zeta Potential and Particle Size Analyzer (Otsuka Electronics, Osaka, Japan). The cells were washed twice with PBS and exposed to SAE at 128, 256, and 512 μg/mL for 15 min. The cells were then washed twice with distilled water and assessed by loading 600 μL of the sample. Cell width measurements were performed based on SEM images. The median area of 30 randomly selected cells per sample was measured. The method of measuring cell permeability using ANS dye was slightly modified. In brief, the cells were grown to the exponential phase at 37 °C in 5 mL LB media and then treated with SAE for 30 min. Stained cells were observed using a fluorescence microscope (Axio Carl Zeiss, Germany).

### *G. mellonella* infection test

*Galleria mellonella* larvae were obtained from SWORM (Cheonan, Republic of Korea). Before injection, healthy *G. mellonella* larvae were starved to increase susceptibility to infection by suppressing the immune response for 3 h at 25 °C^16^. For the positive control, *A. baumannii* ATCC 17978 cells were washed with PBS and diluted to 10^6^ CFU/larva^17^. The same volume of PBS was injected for the negative control. Based on the results of in vitro experiments, the concentration of PMB was set at 0.5 μg/mL/larva and that of SAE was set at 64 μg/mL larva. The larvae were then placed on ice and injected in the last left proleg with 10 µl of Lab-WT strain (10^6^ CFU) with-/-without SAE using 31-gauge 6 mm syringes (BD, USA). A total of 25 *G. mellonella* larvae per condition were then tested after inoculation. The larvae were incubated in Petri dishes (SPL Life Sciences, Republic of Korea) containing 100 mg of wheat bran powder (MG Natural, Republic of Korea) at 37 °C. Larva death was scored for every 24 h by inspecting melanization and the lack of movement in response to touch^16^.

### Determination of PMB susceptibility using osmoprotectant

Osmotic stress reduction experiments were conducted using glycine betaine and trehalose in the same manner as the growth test, killing assay, and induction test. Then, the cells were treated with PMB (4 µg/mL) and SAE (128 µg/mL) with glycine betaine (0.2 mg/mL) or trehalose (0.2 mg/mL). Cells were harvested at each time point and washed twice with PBS, following which 100 μL aliquots were transferred onto LB agar plates. The plates were incubated at 37 °C overnight. Moreover, in the induction test, the cells were harvested using glycine betaine or trehalose at each time point (0, 30, 60, and 120 min), washed twice with PBS, and post-treated with PMB (0.5 μg/mL).

### Identification of active substance in SAE

SAE was analyzed using Quadrupole time-of-flight liquid chromatography–mass spectrometry (Q-TOF–LC/MS) to detect active compounds. SAE in 70% ethanol was loaded in column chromatography using the Sephadex^®^ LH-20 column (1.0 × 60 cm) at room temperature and the loaded SAEs were fractionized using chloroform as a mobile phase. Q-TOF–LC/MS was operated using an Agilent Infinity 1290 UHPLC connected to an Agilent Eclipse Plus C-18 column (2.1 × 100 mm, 2.1 μm) coupled to an Agilent 6550 QTOF mass spectrometer^18^. The injection amount was 1 μL, and the temperature of the column was maintained at 40 °C. The mobile phases consisted of water (A) and acetonitrile (B). Mobile phase A was supplemented with 10 mM ammonium acetate. A gradient of 5–90% B was used for 25 min at a flow rate of 0.3 mL/min. MS was performed using an Agilent 6550 QTOF mass spectrometer equipped with an AJS ESI interface using the following operation parameters: polarity, positive; gas temperature, 250 °C; nebulizer, 35 psig; capillary, (+) 4000 V; and MS and MS/MS range, 100–1500 m/z.

### Antimicrobial susceptibility testing using the active substance

The microdilution checkerboard method was used to assess the synergistic effect of combination treatment with the active substance and PMB^18^. Overnight cultured *A. baumannii* cells was diluted 1/100 in fresh LB broth, and 10^6^ CFU/mL cells that reached the exponential phase were placed in 96-well plates. 6B2N was dissolved in EtOH and the concentration was set at 1–256 µg/mL. The FIC index was calculated using the same methods previously described.

### Quantitative RT-PCR analysis using 6B2N

Overnight cultured *A. baumannii* cells were diluted (1/100) into a fresh LB media and further cultivation was done for 3 h to prepare cell concentration (10^6^ CFU/mL). Freshly grown *A. baumannii* cells were treated with PMB (0.5 μg/mL) and/or 6B2N (32 μg/mL) for 15 min at 37 °C. After each treatment, RNA isolation was performed using the RNeasy Mini Kit (Qiagen, Germany). RNA concentrations were estimated using Nanophotometer N60 (IMPLEN, Germany). The *pmrC* gene was amplified using the following primers: *pmrC*_F (5′-TGGGTAGTCATGGACCTGCAT-3′) and *pmrC*_R (5′-GTTTGCGAACAGCCCTGTATC-3′). To the PCR mixture, iQ SYBR Green Supermix (Bio-Rad, USA) (12.5 μL), each primer (1 μL), and cDNA (1.5 μL) were added to a total volume (25 μL). The thermal program was as follows: 1 cycle of 10 min at 95 °C and 40 cycles of 15 s at 95 °C and 1 min at 60 °C). The quantitative RT-PCR experiment was performed in triplicate, and expression levels of each gene were normalized by the 16S rRNA genes^19^.

### Confocal laser scanning microscopy (CLSM) and Bradford assay

Overnight cultured *A. baumannii* was diluted 1/100 in fresh LB broth, and 10^6^ CFU/mL cells that reached the exponential phase were used. Then, the cells were exposed to PMB (0.5 μg/mL) and 6B2N (16 μg/mL) for 15 min. CLSM images were obtained to confirm the same killing effect by the synergistic action of 6B2N with PMB (the original MIC of PMB alone was 2 μg/mL). The treated samples were assessed using the SYTO9™ green-fluorescent nucleic acid stain (Thermo Fisher Scientific, USA) and Propidium Iodide™ stain (Thermo Fisher, USA). CLSM was performed using LSM 770 (Carl Zeiss Microscope, Jena, Germany). The obtained CLSM images were analyzed and modified using the Zen 3.1 software (Blue edition; Carl Zeiss, Germany). To quantify the protein concentration in the supernatant, all the samples were standardized using the Bradford assay (Bio-Rad Laboratories, Protein Assay Dye Reagent), and triplicates were used. The cells were treated with SAE for 30 min. Stained cells were observed using LSM 770 (Carl Zeiss Microscope, Jena, Germany).

## Results

### Synergistic effects of SAE with PMB

**Figure 1:**
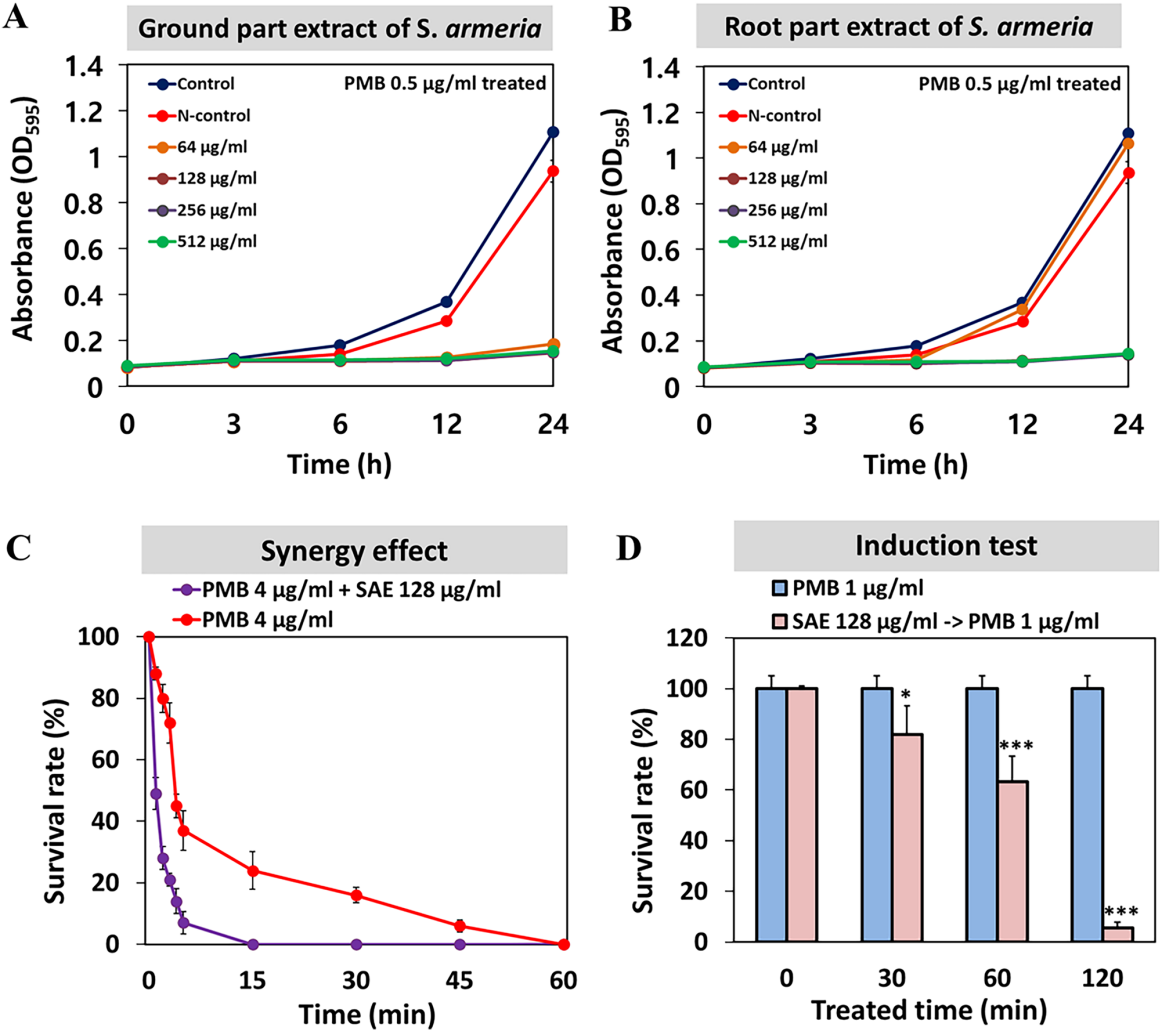
Growth and killing assay of *A. baumannii* treated with PMB and *S. armeria* extract. (**A**) Synergistic effect with ground part extract of *S. armeria*. The concentration of PMB used in the experiment was fixed at 0.5 μg/mL. The same volume of DMSO was used as the positive control. (**B**) Synergistic effect with root part extract of *S. armeria*. (**C**) Bactericidal effect on *A. baumannii* cells treated with only PMB or with a combination of PMB and SAE. (**D**) PMB susceptibility testing by exposure time to SAE. Exposure to SAE (128 μg/mL) for 0, 30, 60, and 120 min was followed by treatment with PMB (1 μg/mL). Statistical analysis was performed using Student’s t-test (*P < 0.05, ***P < 0.005).

A total of 120 natural extracts were screened to investigate their synergistic effects with PMB using the checkerboard assay (Table S1). Of all the tested natural extracts, *Silene armeria* extract with 70% ethanol (hereinafter referred to as SAE) showed a synergistic effect in the presence of PMB for killing *A. baumannii* strain ATCC 17978. In liquid MIC assays, the in vitro activity of PMB in combination with SAE increased even when a lesser amount of PMB was used (0.5 μg/mL) (Table S2). However, the growth of *A. baumannii* was not inhibited by SAE alone (up to 1024 μg/mL) (Fig. S1). Interestingly, when the same low concentration of PMB (0.5 μg/mL) was used, SAE (64 μg/mL) from the parts of the plant above the ground surface (ground parts) was found to be more effective than SAE (128 μg/mL) from the underground (root) parts. This finding suggests that active ingredients that react with PMB are more prevalent in the ground parts, probably because of the presence of many bioactive chemicals in leaves (Fig. 1A,B). All further experiments were therefore conducted using SAE from the ground parts. In addition to the checkerboard assay, a killing assay using a high concentration of PMB (2× MIC) was performed to measure the efficiency of the adjuvant effects of SAE. The survival rate of *A. baumannii* cells was ~ 85% with PMB treatment alone; however, a severe decrease in the survival rate (50%) occurred on 1-min treatment with a combination of PMB and SAE (Fig. 1C). All the cells were dead within 15 min of exposure to the combination; however, this effect could not be observed with PMB treatment alone (Fig. 1C). Pretreatment of cells with SAE for 30 min could sensitize them to PMB (1 μg/mL), indicating that SAE affected the cellular physiology such that the exposed cells were more vulnerable to PMB. These induction tests were conducted after washing SAE-treated or control cells thoroughly with phosphate-buffered saline (PBS). Longer exposure to PMB (> 120 min) considerably decreased the survival rate (12%) of *A. baumannii* cells. This finding suggested that continued cellular changes appeared in SAE-treated cells; however, the same concentration of SAE alone was insufficient to kill the cells (Fig. 1D, Table S2).

### Changes in morphology and pathogenicity following combination treatment

**Figure 2:**
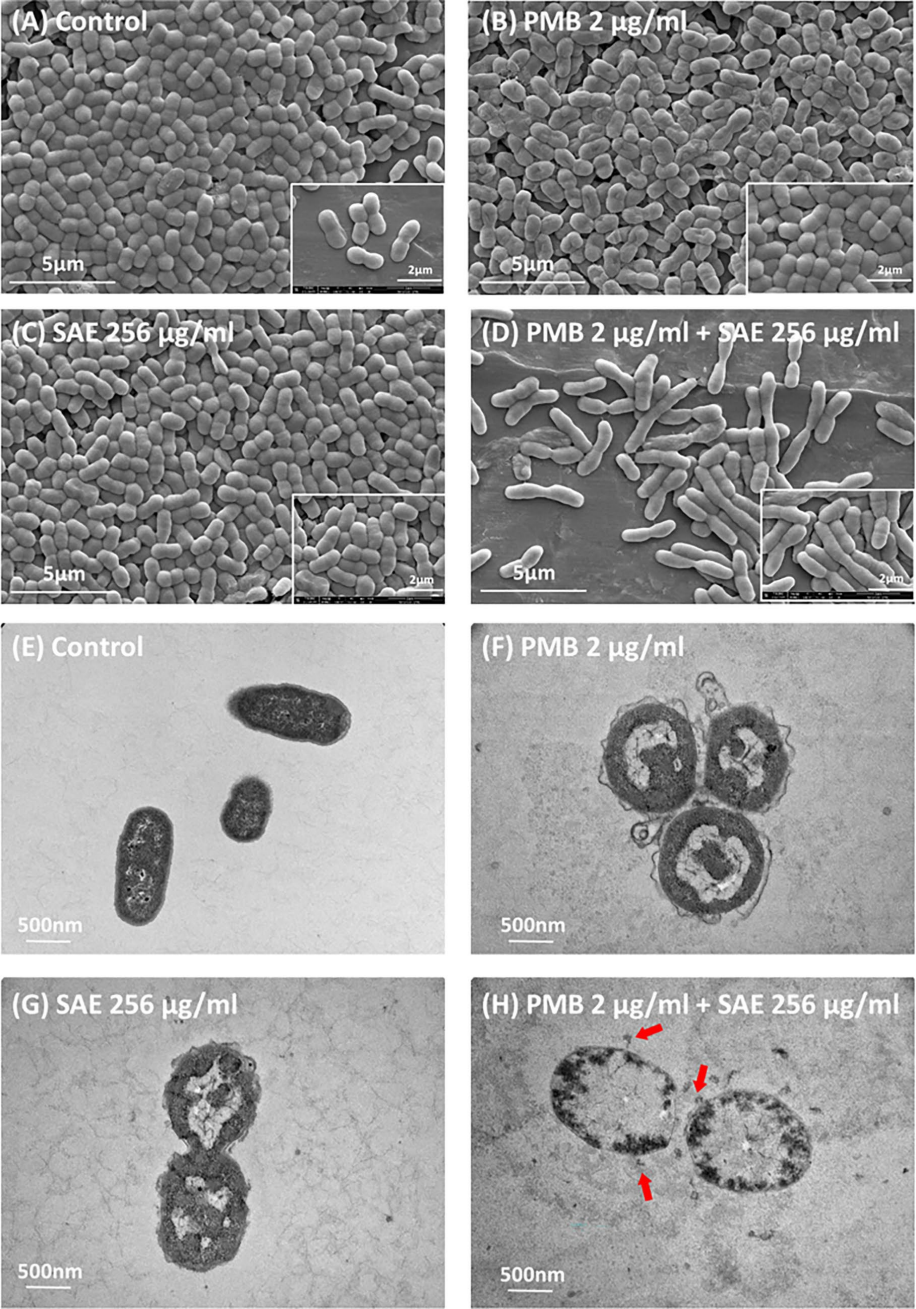
SEM and TEM of *A. baumannii* ATCC17978 cells treated with PMB and SAE. (**A**) SEM image of *A. baumannii* treated with DMSO as the control. (**B**) PMB for 15 min at a concentration of 4 µg/mL. (**C**) SAE for 15 min at a concentration of 256 µg/mL. (**D**) *A. baumannii* cells treated for 15 min with a combination of PMB at a concentration of 4 µg/mL and SAE at a concentration of 256 µg/mL. (**E**) TEM image of *A. baumannii* treated with DMSO as the control. (**F**) PMB for 15 min at a concentration of 1 µg/mL. (**G**) SAE for 15 min at a concentration of 256 µg/mL. (**H**) *A. baumannii* treated for 15 min with a combination of PMB at a concentration of 2 µg/mL and SAE at a concentration of 256 µg/mL. Red arrows indicated membrane blebbing under a combination of PMB and SAE.

Morphological changes in SAE-treated *A. baumannii* cells were observed by scanning electron microscopy **(**SEM) and transmission electron microscopy (TEM) (Fig. 2). SEM images revealed that cellular swelling occurred when the cells were exposed to either PMB or SAE for 15 min (Fig. 2A–C). The swelling was slightly more severe under SAE-induced conditions. Moreover, the combination treatment made the cells longer than the other treatments (Fig. 2D). Measurement of the width of 30 cells under each condition confirmed a 60% increase in cell width (average, 0.42 μm vs. 0.67 μm) following SAE treatment; however, the same concentration of SAE could not affect the viability of SAE-treated cells (Fig. S2A). Control cells had smooth surfaces and condensed chromosomes, while PMB-treated or SAE-treated cells had irregular shapes with bleb formation on the surface (Fig. 2E–H). TEM revealed that both PMB and SAE caused dramatic chromosomal relaxation, probably because of ATP loss, and consequently reduced gyrase activity because of increased membrane permeability (Fig. 2)^20^. Treatment with a combination of PMB and SAE resulted in severely damaged cellular membranes, causing the release of cellular components into the media and membrane blebbing under this synergistic condition (Fig. 2H, marked with red arrows).

**Figure 3:**
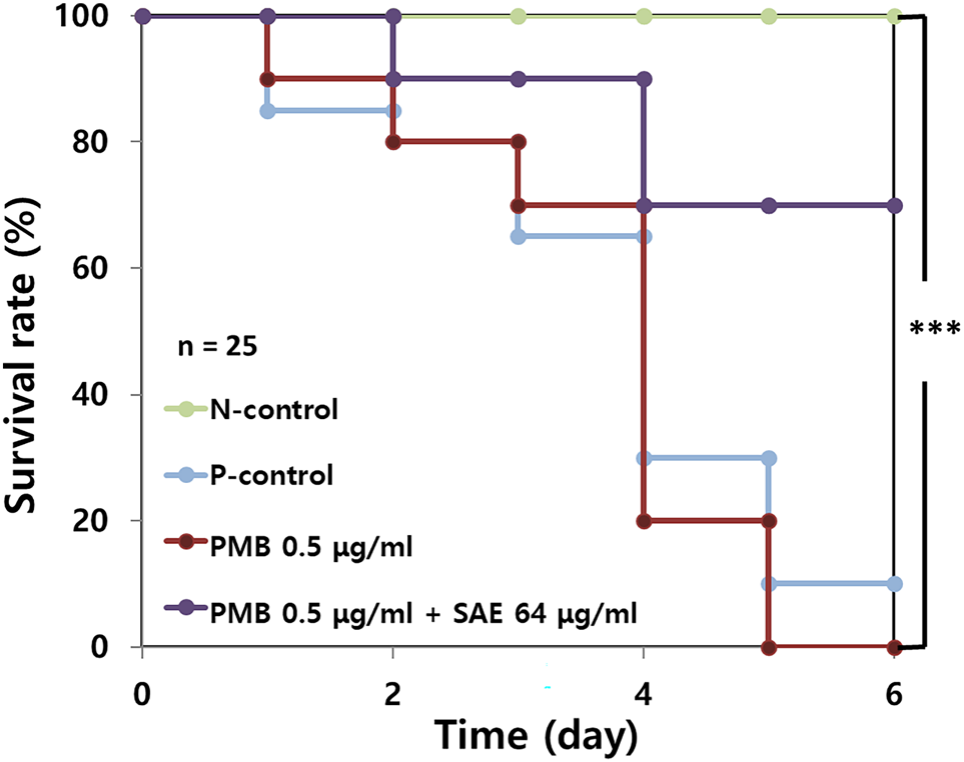
In vivo synergistic effect using *G. mellonella*. *G. mellonella* was divided into two groups: (1) PMB alone and (2) PMB and SAE. A total of 25 *Galleria mellonella* larvae per condition were infected with the *A. baumannii* ATCC 17978 to compare their pathogenicity in vivo. The survival rate of each group was assessed for 5 days. Statistical analysis was performed using Student’s *t*-test (*P < 0.05, ***P < 0.005).

The measured zeta potential values of SAE-treated cells displayed a tendency in which the surface charge of the cells could be more negative under SAE-treated conditions; however, the magnitude of zeta potential change suggested that the significance of this measurement was low because little change was observed when a low concentration of SAE (128 μg/mL) was used (Fig. S2B). More negative zeta potential values with SAE treatment indicated the possibility of increased binding of PMB (a cationic polypeptide antibiotic) to the cells. The addition of 8-anilinonaphthalene-1-sulfonic acid (ANS) to SAE-treated cells caused a dramatic increase in the fluorescence intensity, resulting in increased cellular permeability with an increase in the concentration of SAE (Fig. S2C). Our data indicated that SAE could change membrane permeability without affecting cellular viability, which may promote the cellular uptake of PMB and consequently increase membrane and cellular macromolecule damage, eventually leading to cell death. The *G. mellonella* infection model was used to confirm the in vivo synergistic effects of SAE with PMB. *G. mellonella* cells (n = 25) infected with *A. baumannii* ATCC 17978 with/without a low concentration of PMB (0.5 μg/mL) were dead within 5 days. However, the addition of SAE resulted in a 75% survival rate of larvae within the same periods, indicating that treatment with a combination of PMB and SAE could reduce the amounts of PMB required for therapeutic purposes (Fig. 3).

### Reduction of synergistic effect with glycine betaine and trehalose

Cellular swelling by SAE and by combination treatments led us to speculate that damaged membranes could cause the accumulation of damaged proteins because of the loss of ATP and change in membrane integrity under our study conditions. Several osmolytes, such as glycine betaine and trehalose, can stabilize proteins and can prevent protein denaturation^21^. The growth of *A. baumannii* cells was completely inhibited by the combination treatment; however, this could not be seen in the presence of either glycine betaine or trehalose (Fig. S3). Partial growth recovery occurred in the presence of a low concentration of glycine betaine (0.1 mg/mL) under PMB–SAE (256 μg/mL) conditions; however, a higher concentration of glycine betaine (0.2 mg/mL) was required to restore the growth of *A. baumannii* cells under PMB–SAE (512 μg/mL) conditions (Fig. S3B). In the case of trehalose, a low concentration (0.1 mg/mL) could not recover cell growth, but an increased concentration (0.2 mg/mL) restored the growth to a similar degree to that restored by glycine betaine (Fig. S3C,D). High concentrations of PMB (4 μg/mL) resulted in cell death within 60 min; these cells could not be recovered by either glycine betaine or trehalose (Fig. S4A,B). Pre-exposure of cells to glycine betaine or trehalose could cancel the synergistic effect of SAE with PMB, suggesting that these compounds play an indirect role by modulating the transcriptional levels of unknown defense genes rather than directly binding to PMB or damaged proteins (Fig. S4C,D).

### Identification of active compounds in SAE

**Figure 4:**
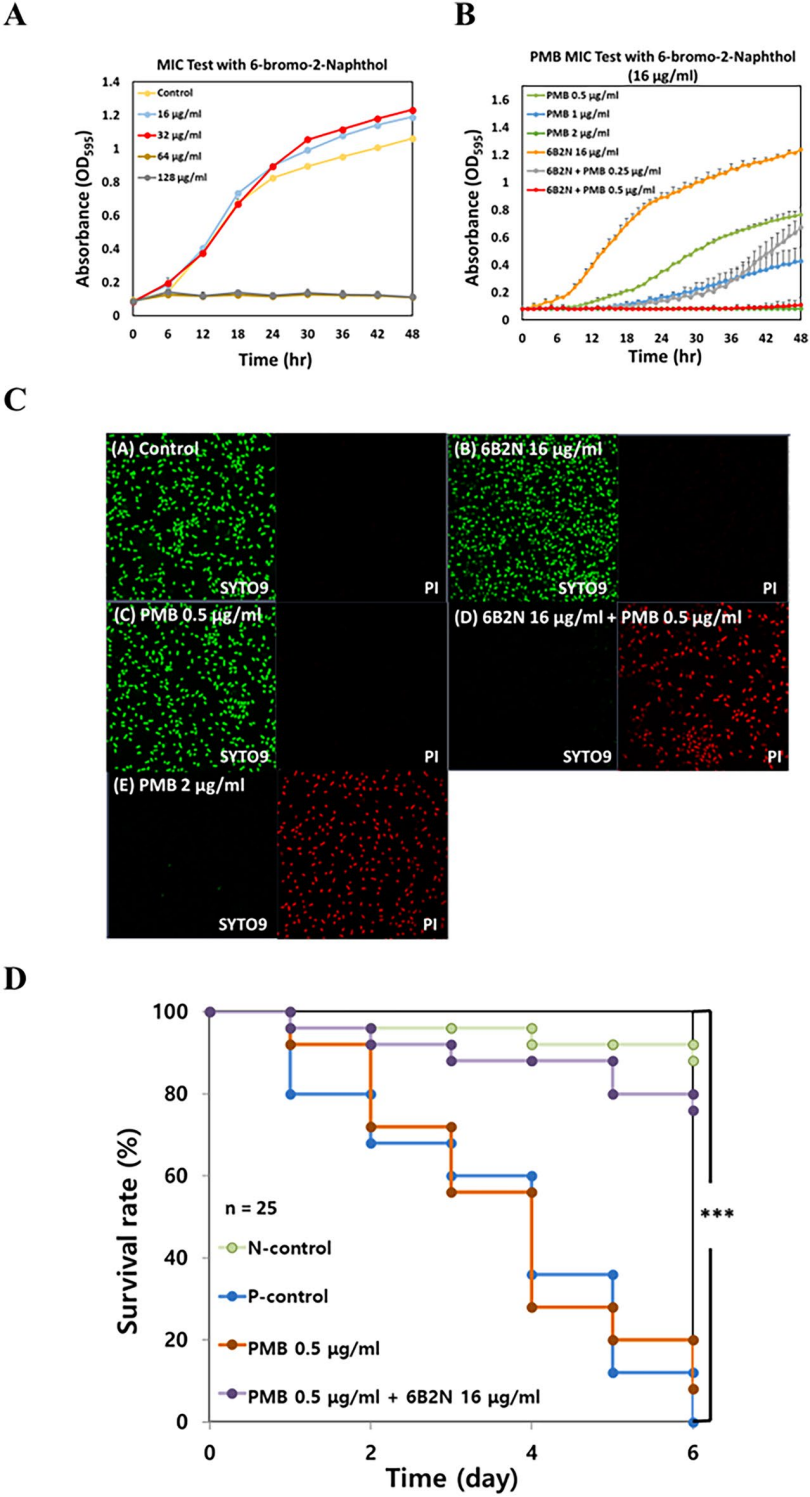
Bactericidal effect of 6-bromo-2-naphthol on *A. baumannii*. (**A**) Growth test of *A. baumannii* treated with only 6-bromo-2-naphthol. The concentration of 6-bromo-2-naphthol was set at 1–256 μg/mL. (**B**) Assessment of the synergistic effect of PMB and 6-bromo-2-naphthol. The concentration of 6-bromo-2-naphthol used in the experiment was fixed at 16 μg/mL. The same volume of ethanol was used as the positive control. (**C**) Visualization of synergistic effects using SYTO9™ and PI dye based on CLSM images. (**D**) In vivo synergistic effect using *G. mellonella*. *G. mellonella* larvae were divided into two groups: (1) PMB alone and (2) PMB and 6B2N. A total of 25 larvae of *Galleria mellonella* per condition were infected with the *A. baumannii* ATCC 17978 cells in the presence and absence of our tested compounds*.* The survival rate of each group was assessed for 5 days. Statistical analysis was performed using Student’s *t*-test (*P < 0.05, ***P < 0.005).

To examine the active components in SAE, it was divided into fractions using different types of solvents (hexane, chloroform, and ethyl acetate) and distilled water was used as a control. Each dissolved fraction was tested with PMB (0.5 μg/mL) to identify the fractions effective as synergistic adjuvants with PMB. Only the chloroform fraction of SAE was valid for combination treatment with PMB to inhibit cell growth (Fig. S5A). Q-TOF–LC/MS was performed using the chloroform fraction from SAE to identify the active ingredients. Literature search and chemical availability data led us to test 6B2N as a possible candidate as an adjuvant with PMB. 6B2N, which is a member of the naphthol class, has a hydroxyl group, and is more reactive than phenols, was selected as the synergistic active substance; its molecular weight was 280.98 m/z (Fig. S5B). The formula of this peak was C_12_H_10_BrO_3_, which is 6B2N combined with acetate derived from ammonium acetate included in a solvent of Channel A. The UV/Vis spectrum showed a significant peak at 200–300 nm (Fig. S5C). Based on the mass spectrum peak results, a fragmentation pathway of 6B2N combined with acetate was proposed (Fig. S5D). To confirm the synergistic effect of 6B2N with PMB based on Q-TOF–LC/MS, the cell growth curve of *A. baumannii* treated with 6B2N was assessed. The cells were completely killed with only 6B2N (64 μg/mL) (Fig. 4A). The bactericidal effect was synergistically enhanced when the bacteria were treated with a combination of 6B2N (16 μg/mL) and PMB (0.5 μg/mL) (Fig. 4B). The SYTO9™ or Propidium Iodide™ (PI) was added to cells with SAE (16 μg/mL) or PMB (0.5 μg/mL), which demonstrated that SAE or low concentration of PMB alone, could not affect the viability of cells (Fig. 4C, panel A–C). However, combinatory treatment with 6B2N (16 μg/mL) and PMB (0.5 μg/mL) caused a dramatic increase in PI fluorescence intensity, showing their synergistic killing effect on *A. baumanii* cells (Fig. 4C, panel D). The same killing effect was also observed using the MIC (2 μg/mL) of PMB (Fig. 4C, panel E). Live/dead cell image analyses also proved this synergistic bactericidal effect on *A. baumannii* cells. The *G. mellonella* infection model was used to confirm the in vivo synergistic effects of 6B2N with PMB. *G. mellonella* larvae (n = 25) infected with *A. baumannii* ATCC 17978 cells with/without a low concentration of PMB (0.5 μg/mL) were dead within 6 days. However, the addition of 6B2N resulted in a 76% survival rate of larvae within the same periods, indicating that treatment with a combination of PMB and 6B2N could reduce the amounts of PMB required for therapeutic purposes (Fig. 4D).

### Mechanisms of a synergistic effect of 6B2N

**Figure 5:**
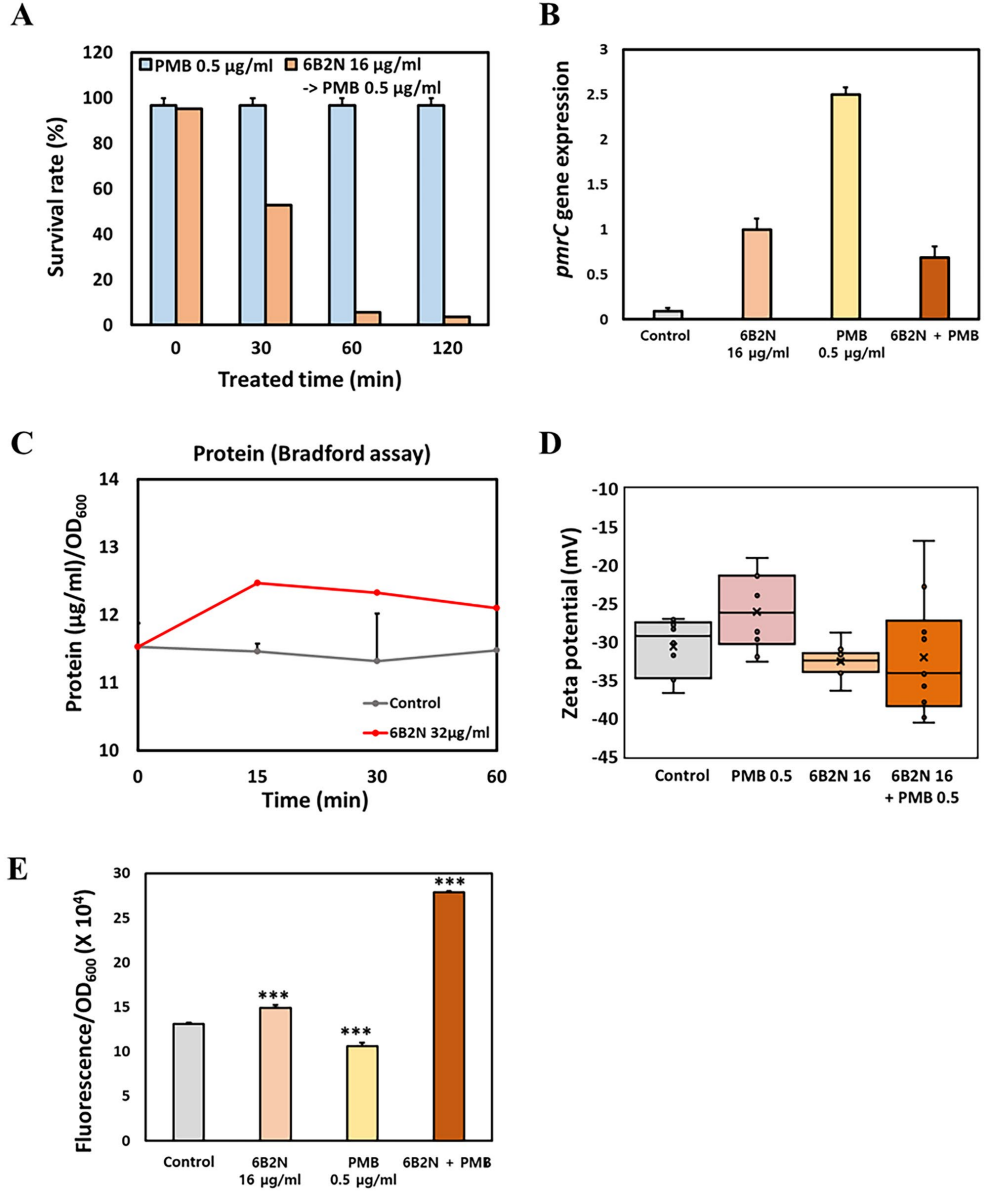
Mechanisms of synergistic effect on *A. baumannii* ATCC17978 treated with 6-bromo-2-naphthol. (**A**) Induction test using 6-bromo-2-naphthol. (**B**) Changes in the expression level of *pmrC*. (**C**) The concentration of protein in the supernatant of *A. baumannii* treated with only 6B2N. The concentration of 6B2N was set at 16 μg/mL. (**D**) The surface zeta potential of *A. baumannii* after treatment with 6B2N. (**E**) Assessment of changes in cellular permeability using ANS dye. Statistical analysis was performed using Student’s t-test (*P < 0.05, ***P < 0.005).

Pretreatment of cells with 6B2N for 30 min could sensitize them to PMB (0.5 μg/mL), indicating that 6B2N affected the cellular physiology such that the exposed cells were more vulnerable to PMB (Fig. 5A). Longer exposure to 6B2N (> 60 min) considerably decreased the survival rate (6%) of *A. baumannii* cells. This finding suggested that continued cellular changes appeared in 6B2N-treated cells; however, the same concentration of 6B2N alone was not sufficient to kill the cells. We assumed that 6B2N could change the membrane surface charge, which promoted the cellular uptake of PMB. The addition of only 6B2N decreased the expression of *pmrC* in the *pmrCAB* operon, conferring binding affinity to PMB (Fig. 5B)^22^. The observed synergistic effects with 6B2N may be attributed to increased binding to PMB caused by lower *pmrC* expression. SAE induced chromosomal relaxation and membrane damage in bacterial cells, which may have resulted in the leakage of intracellular substances, such as proteins. The extracellular protein amounts in the supernatant were higher in the 6B2N-treated cells than in the control cells (Fig. 5C). The zeta potential values also indicated that the surface charge of the cells could be more negative under 6B2N-treated conditions (Fig. 5D). More negative zeta potential values with 6B2N treatment indicated the possibility of increased binding of PMB (a cationic polypeptide antibiotic) to the cells. More importantly, the cells treated with 6B2N and ANS dye had higher membrane permeability and displayed greater fluorescence intensity, as seen in our SAE-treated samples (Fig. S2C, Fig. 5E).

Assessment of the possible synergistic effect of 6B2N with other antibiotics (e.g., meropenem, doxycycline, gentamicin, and erythromycin) revealed that antibiotics working inside the cytosol exhibited a synergistic effect with 6B2N; however, this effect was not observed with the cell wall-acting antibiotics meropenem and ampicillin (Table S3). The combination of 6B2N and PMB was more effective (1/4 MIC) than that of 6B2N any other antibiotic (1/2 MIC). Moreover, the adjuvant effects of 6B2N were assessed using 20 other strains (five bacterial species and 15 *A. baumannii* clinical isolates). Interestingly, adjuvant effects were observed in nine out of 15 *A. baumannii* isolates, but not other species, which indicated that the specificity of 6B2N was only to some *A. baumannii* strains (Table S4).

## Discussion

Combinations of natural plant extracts or other secondary metabolites with existing antibiotics are effective in treating antibiotic-resistant bacterial infections^23^. PMB could have a synergistic effect with netropsin, a secondary metabolite of *Streptomyces*, to kill *A. baumannii* and *E. coli*^24^. Essential oils and various leaf extracts of *S. armeria* have been shown to inhibit the growth of food spoilage-related and foodborne pathogens because of the presence of antibacterial substances, such as coumarin and caryophyllene oxide^25^. In the present study, SAE was found to have a synergistic effect with PMB to kill many tested *A. baumannii* strains, possibly because of SAE-mediated membrane perturbation (Fig. 2). When bacterial cells were exposed to chemicals present in SAE, the water activities inside and outside the cells were possibly lowered; this finding may be linked to cellular swelling (Fig. 2). Cells subjected to low water activities showed altered morphologies by being larger and rounder and tended to stick together to a greater extent^26^. Furthermore, increased salinity resulted in a higher proportion of anionic lipids (than neutral lipids), such as cardiolipin, in *A. baumannii* cells^27^. SAE probably changed the lipid composition to increase the negative charge on the cell wall surface for increased binding to PMB. As natural plant extracts are complex mixtures of metabolites, it was difficult to pinpoint the most active constituents among the many possible candidates.

Q-TOF–LC/MS suggested that several candidate compounds (e.g., 6B2N, linoleic acid, piperochromeoic acid, and pregna-4,16-diene-3,11,20-trione) could be considered as adjuvants for potential combination therapy with PMB. Linoleic and oleic acids from *H. pedunculatum* are known to have antimicrobial activities against only gram-positive bacteria, including *S. aureus*^28^. Pregna-4,16-diene-3,11,20-trione was excluded by us from further analyses because it has been proven to have higher antiandrogenic activity in the hamster prostate^29^. Of the selected candidates, 6B2N was the only promising adjuvant that was experimentally tested to have a synergistic effect with PMB (Fig. 4). A structurally similar chemical, 1-naphthol, also had a synergistic effect with PMB (0.5 μg/mL) (Fig. S6). The synergistic concentration of 6B2N (16 μg/mL) was twofold lower than that of 1-naphthol (32 μg/mL), indicating that 6B2N dramatically affected the membranes of *A. baumannii* cells (Table S5). Several modes of action may be simultaneously involved in our combination treatment (e.g., the inhibition of multiple targets, changes in physicochemical interactions, and obstacles resulting from antibacterial resistance mechanisms^30^. PMB is known to destabilize bacterial LPS through charge interaction, which leads to cell death^31^. Destabilization of the LPS layer allows the penetration of more PMB into the periplasm, essentially providing a self-promoted uptake pathway for PMB to reach the cytoplasmic membrane^32^. 6B2N may contribute to. Our findings suggest that 6B2N from *S. armeria* can serve as a new synergistic compound for use with antibiotics by increasing cell membrane permeability and destabilizing the LPS layer of *A. baumannii* cells, thereby enhancing the effect of PMB.

## Supplementary Information

Supplementary Information.

## Data Availability

All relevant data supporting the conclusions of this study are included in this article and its supplementary information file.

## Abbreviations

ANS: 8-Anilinonaphthalene-1-sulfonic acid
6B2N: 6-Bromo-2-naphthol
MDR: Multidrug-resistant
MRSA: Methicillin-resistant *Staphylococcus aureus*
PI: Propidium iodide
PMB: Polymyxin B
SAE: *Silene armeria* extract
