# Supplementary material for: 6-Bromo-2-naphthol from Silene armeria extract sensitizes Acinetobacter baumannii strains to polymyxin
Source: Sci Rep. 2022 May 20;12:8546. doi: 10.1038/s41598-022-11995-y (PMC9123208; doi:10.1038/s41598-022-11995-y)
Supplement: Supplementary file 1 — Supplementary Information. [file 41598_2022_11995_MOESM1_ESM.docx]

**6-Bromo-2-naphthol from *Silene armeria* extract sensitizes *Acinetobacter baumannii* strains to polymyxin**

**Mingyeong Kang^1^, Wonjae Kim^1^, Jaebok Lee^1^, Hye Su Jung^2^, Che Ok Jeon^2^, and Woojun Park^1^***

^1^Laboratory of Molecular Environmental Microbiology, Department of Environmental Science and Ecological Engineering, Korea University, Seoul, 02841, Republic of Korea

^2^Department of Life Science, Chung-Ang University, Seoul, 06974, Republic of Korea

**Running title:** Synergistic effect of 6-bromo-2-naphthol with polymyxin B

**Keywords:** antibiotics; natural extract; pathogen; *Galleria mellonella;* toxicity; susceptibility

***Corresponding author:** Dr. Woojun Park, Department of Environmental Science and Ecological Engineering, Korea University, Seoul 02841, Republic of Korea

**E-mail:** wpark@korea.ac.kr

**Fax:** +82-2-953-0737

**Phone:** +82-2-3290-3067

**Notes:** The authors declare no competing financial interest.

**Supplementary Fig. S1:** Growth of *A. baumannii* treated with ground part extract of *S. armeria*. The same volume of DMSO was used as the negative control. The growth of *A. baumannii* ATCC 17978 was not inhibited on treatment with only SAE even at a concentration of 1024 μg/mL.

**
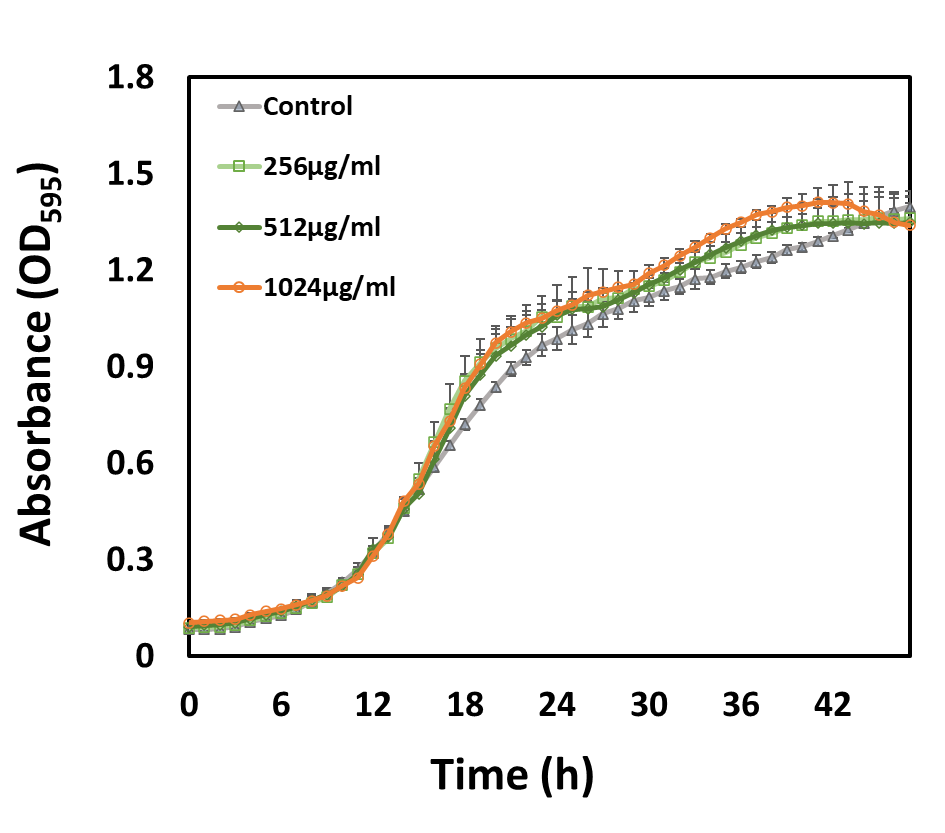
**

**Supplementary Fig. S2:** Changes in cell width and integrity by adjuvant effects of 6-bromo-2-naphthol on *A. baumannii*. (A) Cell width of *A. baumannii* treated with 6-bromo-2-naphthol. The width was measured on the basis of SEM images (n = 30). (B) Changes in cell surface charge of *A. baumannii*. (C) Changes in membrane permeability using ANS dye. Fluorescence values were measured after exposure to each concentration for 30 min.


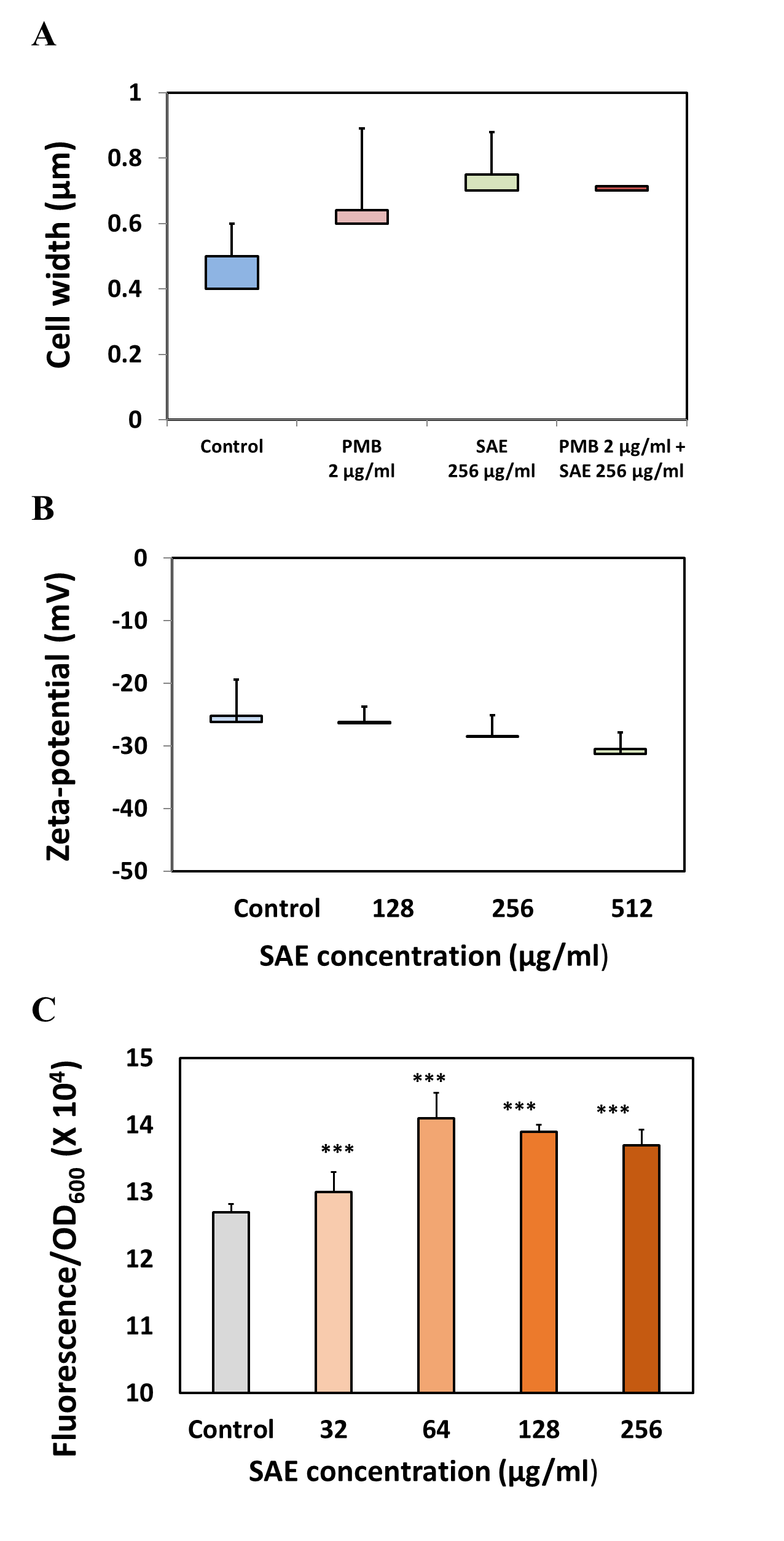


**Supplementary Fig. S3:** Determination of PMB susceptibility using osmoprotectant. (A) Growth curve obtained after treatment with 0.1 mg/mL glycine betaine. (B) Growth curve obtained after treatment with 0.2 mg/mL glycine betaine. (C) Growth curve obtained after treatment with 0.1 mg/mL trehalose. (D) Growth curve obtained after treatment with 0.2 mg/mL trehalose.

**
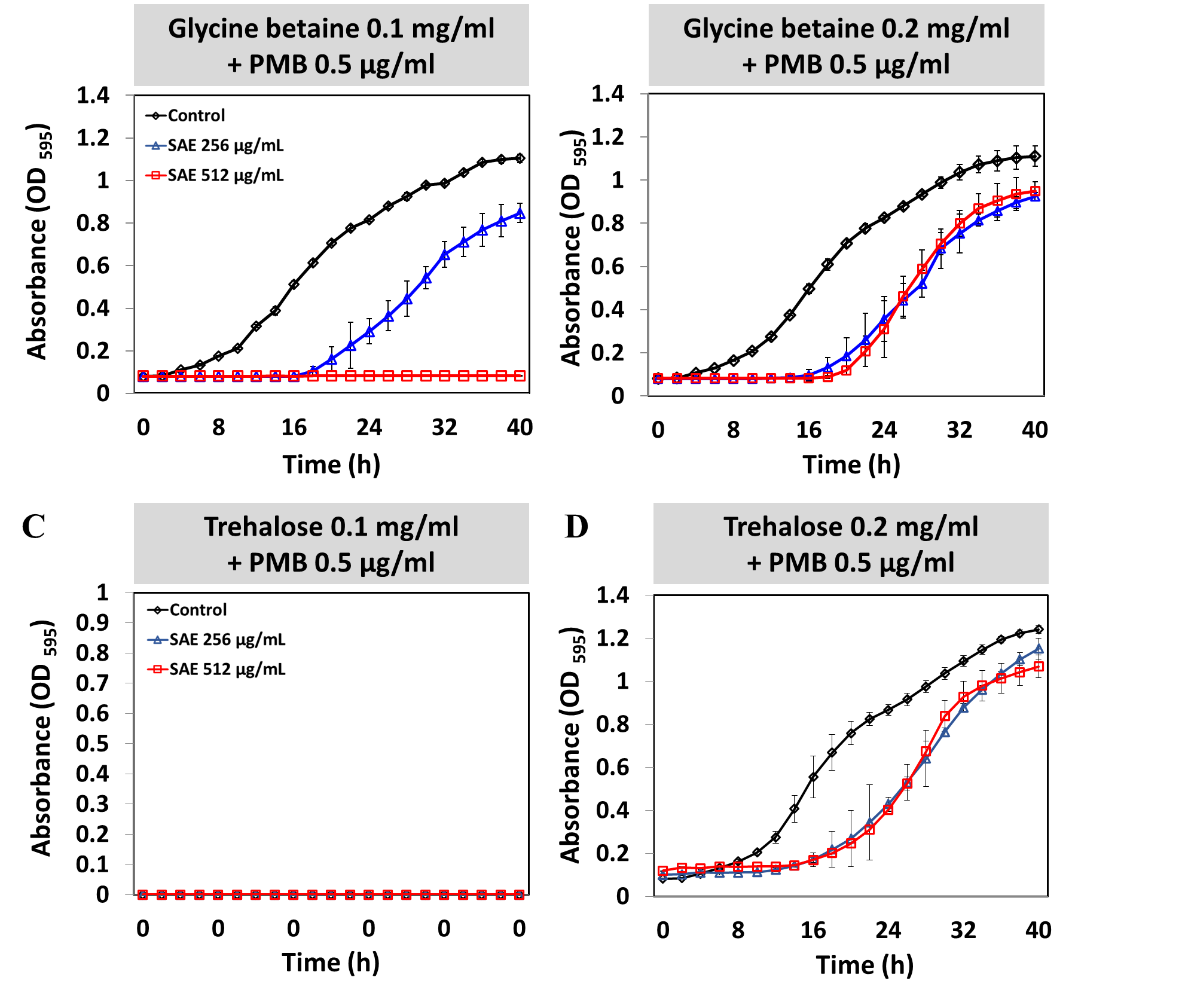
**

**Supplementary Fig. S4:** Bactericidal and induction effects of plant extract with osmoprotectant. (A) Survival rate of *A. baumannii* ATCC17978 exposed to glycine betaine at a concentration of 0.2 mg/mL for 24 h. (B) SAE induction effects of *A. baumannii* ATCC17978 exposed to glycine betaine under the same conditions as those in the killing assay of glycine betaine-treated cells. (C) Survival rate of *A. baumannii* ATCC17978 exposed to trehalose at a concentration of 0.2 mg/mL for 24 h. (D) Induction effects of SAE on *A. baumannii* ATCC17978 exposed to glycine betaine under the same conditions as those in the killing assay of trehalose-treated cells.

**
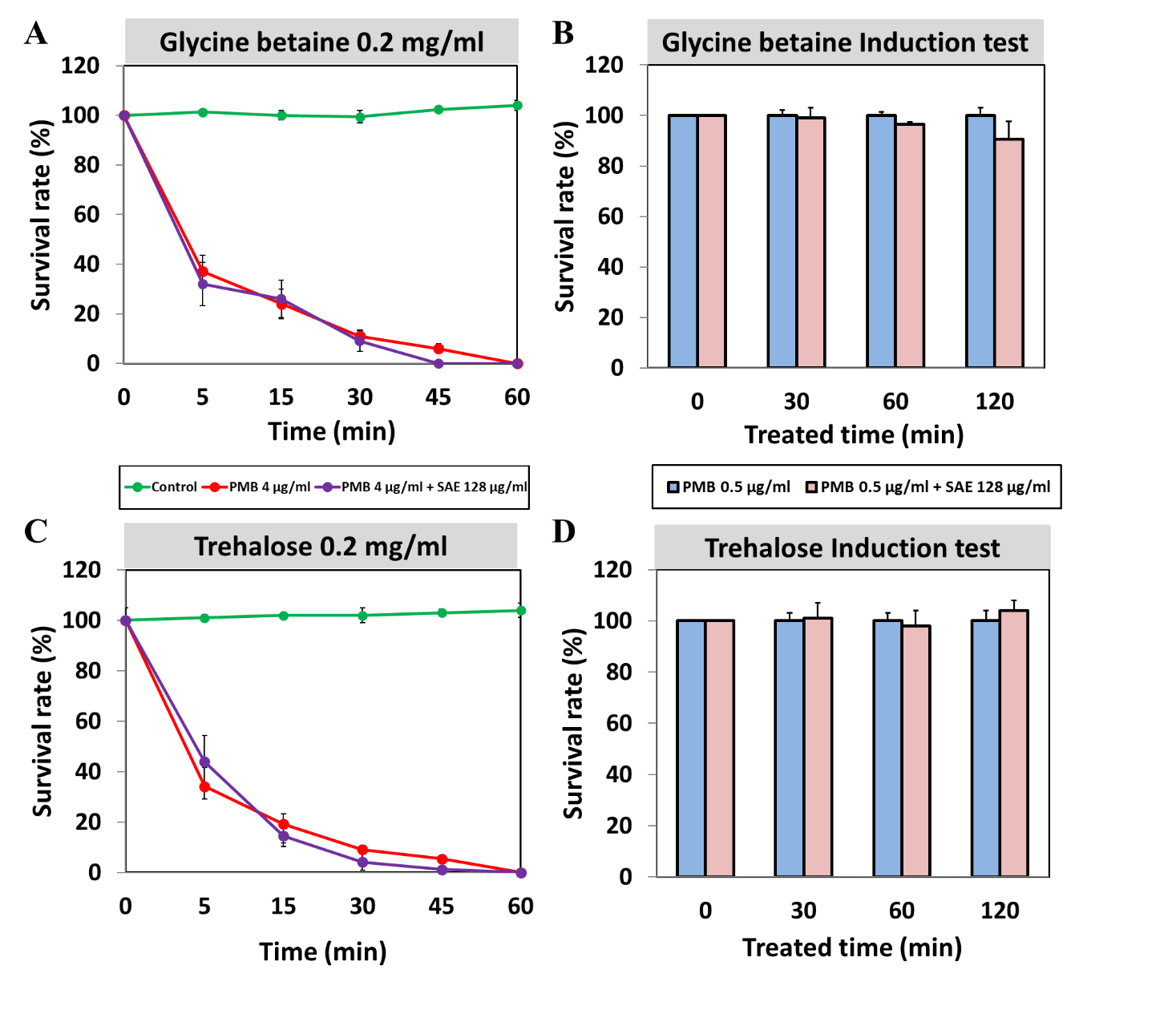
**

**Supplementary Fig. S5:** Extraction and analyses of active substance from natural extract based on Q-TOF–LC/MS. (A) *A. baumannii* ATCC17978 was exposed to the fraction layer (0.5 μg/mL) for 24 h. A synergistic effect was observed only in the chloroform fraction layer. (B) Mass spectrum peak results. (C) The UV/Vis spectrum showed a significant peak at a retention time of 5.8 min. The absorbance values were observed at 200–300 nm. (D) Structural formula of 6-bromo-2-naphthol and the proposed fragmentation pathway of 6-bromo-2-naphthol combined with acetate.

**
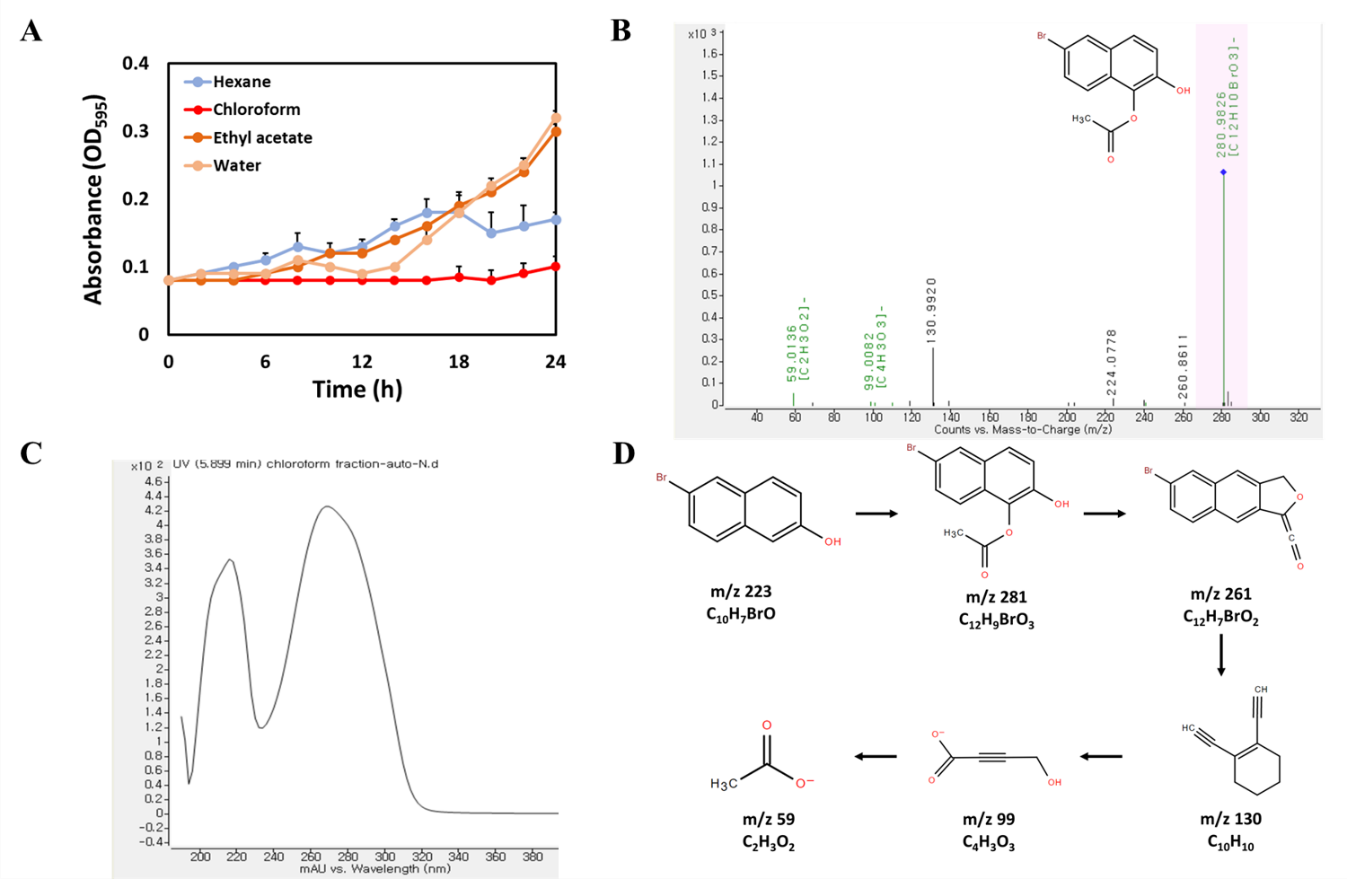
**

**Supplementary Fig. S6:** Growth of *A. baumannii* cells treated with 1-naphthol and polymyxin B. (A) Growth curve of *A. baumannii* ATCC17978 treated with 1-naphthol. The concentration of 6-bromo-2-naphthol was set at 0, 1, 2, 4, 8, 16, 32, 64, 128, and 256 μg/mL to identify bactericidal effect on *A. baumannii*. Cells were completely killed by 1-naphthol at 128 μg/mL. (B) Synergistic effect of 1-naphthol with polymyxin B. The concentration of 1-naphthol used in the experiment was fixed at 32 μg/mL. Cells were completely killed by a combination of 1-naphthol at 32 μg/mL and polymyxin B at 0.5 μg/mL.

**
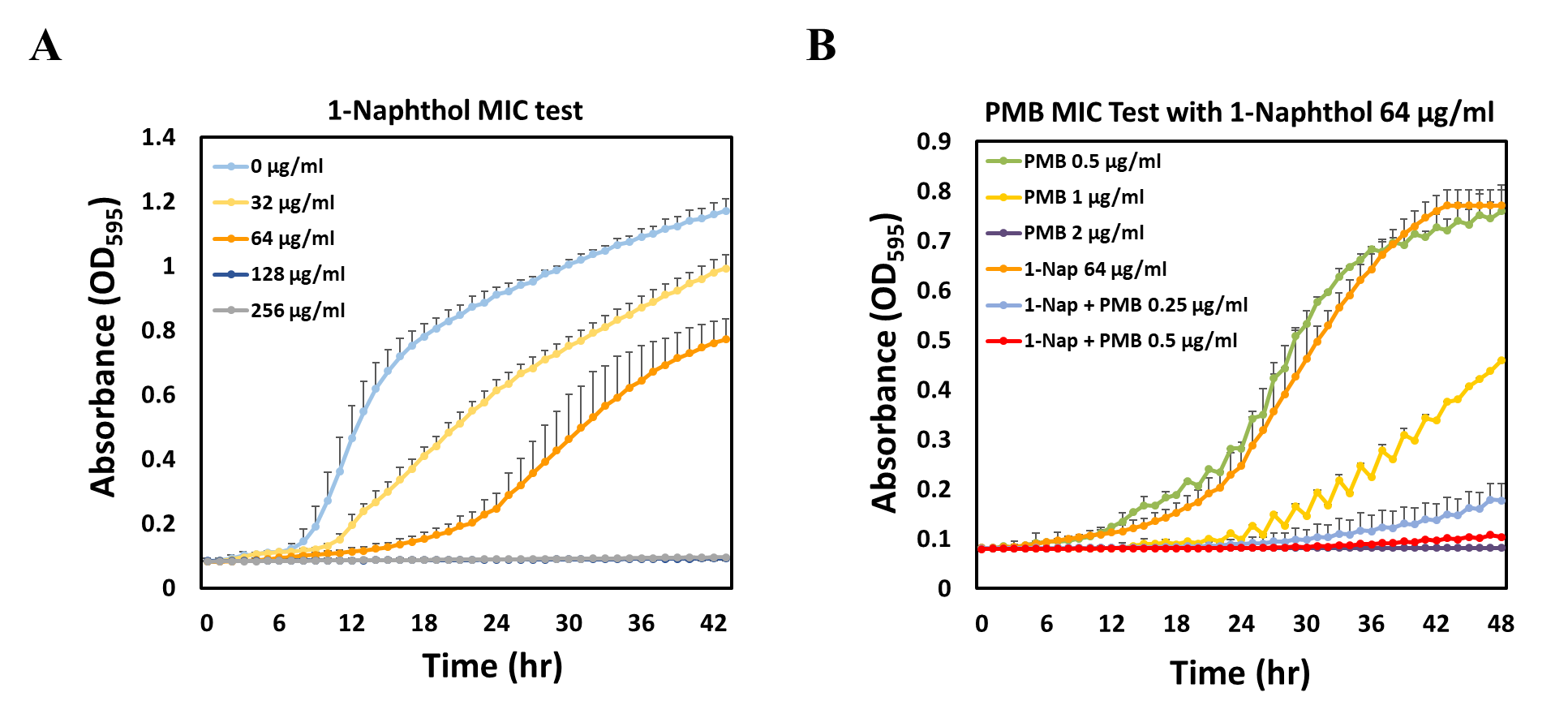
**

**Supplementary Table S1:** List of natural extracts used in the experiment.

**
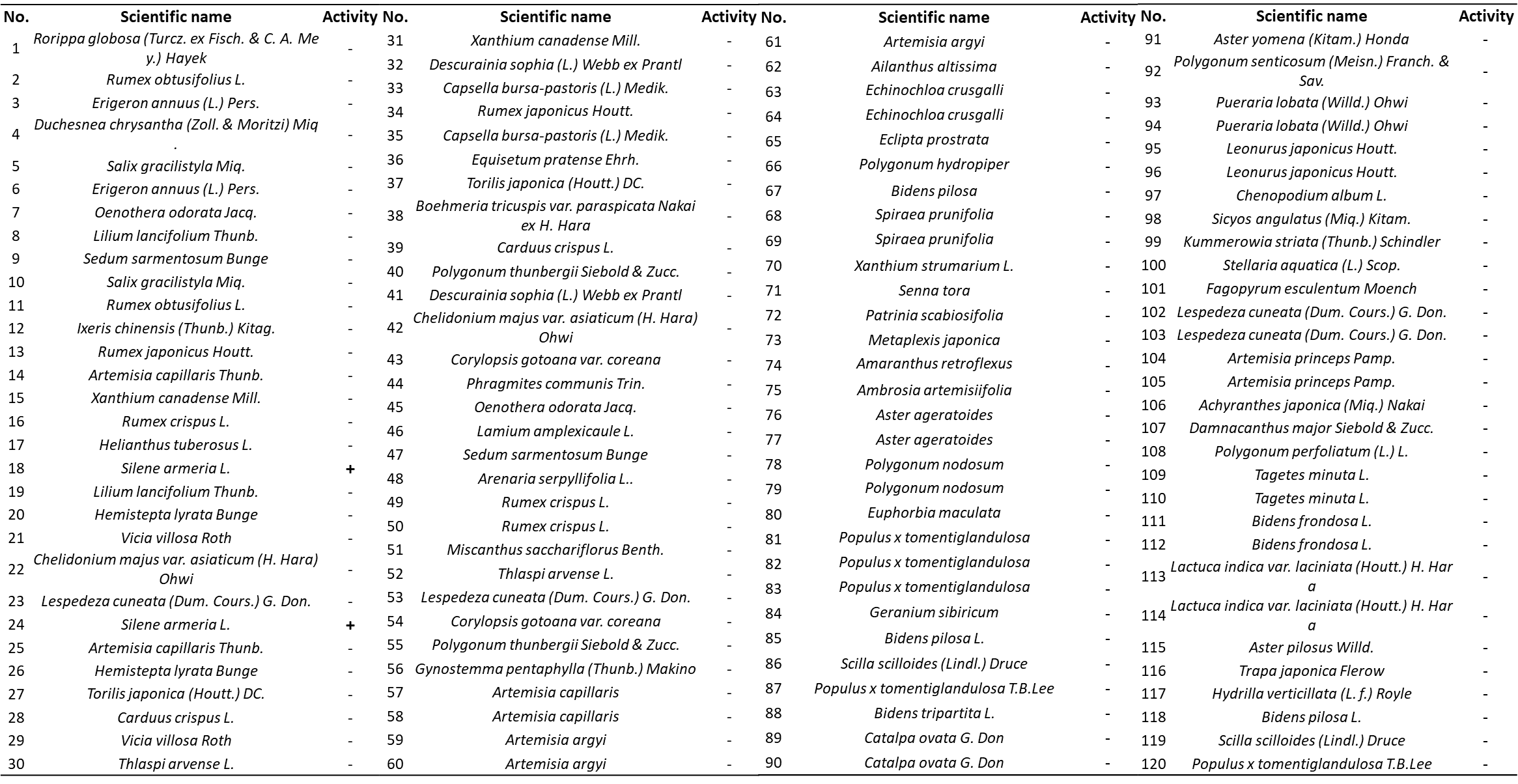
**

**Supplementary Table S2:** Fractional inhibitory concentration (FIC) of PMB and *S. armeria* extract. FIC^a^, The concentration of only PMB in the combination used for FIC was 0.5 μg/mL. FIC^b^, The concentration of only SAE in the combination used for FIC. FIC index, calculated as the sum of FICs of *S. armeria* extract and PMB.

| **Bacteria** | **Agent (polymyxin B)** | **MIC** | | **FIC^a^** | **FIC^b^** | FICI | **Result** |
| --- | --- | --- | --- | --- | --- | --- | --- |
|  |  | Alone | **Combination** |  |  |  |  |
| *Acinetobacter baumannii* ATCC 17978 | *S. armeria* ground part (No. 18) | >1024 | 64 | 0.25 | 0.0625 | 0.31 | Synergy |
|  | *S. armeria* root part (No. 24) | >1024 | 128 | 0.25 | 0.125 | 0.375 | Synergy |

**Supplementary Table S3:** Synergistic effects of 6-bromo-2-naphthol with various antibiotics. Assessment of the possible synergistic effects of 6B2N with other antibiotics, including meropenem, ampicillin, doxycycline, gentamicin, and erythromycin, revealed that most antibiotics, except for meropenem and ampicillin, had a synergistic effect with 6B2N.

| **Antibiotics** | **MIC** | | **Activity** |
| --- | --- | --- | --- |
|  | **Alone** | **Combination** |  |
| Polymyxin B | 2 | 0.5 | + |
| Meropenem | 1 | 1 | − |
| Ampicillin | 256 | 256 | − |
| Doxycycline | 1 | 0.25 | + |
| Gentamicin | 1 | 0.25 | + |
| Erythromycin | 16 | 8 | + |

**Supplementary Table S4:** Antimicrobial testing with polymyxin B and 6-bromo-2-naphthol (6B2N) in other strains. Adjuvant effect tests were performed using 20 strains (five environmental bacteria and 15 clinical isolates). FIC^a^, The concentration of 6B2N used for the fractional inhibitory concentration (FIC) was fixed at 16 μg/mL. FIC^b^, The minimum inhibitory concentration (MIC) of polymyxin B was not determined. FIC index, calculated as the sum of FICs of 6B2N and polymyxin B.

| **Bacteria** | **MIC** | | **FIC^a^** | **FIC^b^** | **FICI** | **Result** |
| --- | --- | --- | --- | --- | --- | --- |
|  | **Alone** | **Combination** |  |  |  |  |
| *Escherichia coli* K12 | 0.25 | 0.25 | 1 | 1 | 2 | − |
| *Acinetobacter oleivorans* DR1 | 0.25 | 0.25 | 1 | 1 | 2 | − |
| *Pseudomonas aeruginosa* PAO1 | 1 | 1 | 1 | 1 | 2 | − |
| *Salmonella typhimurium* LT2 | 4 | 4 | 1 | 1 | 2 | − |
| *Listeria monocytogenes* ScottA | 4 | 4 | 1 | 1 | 2 | − |
| **Clinical isolates** |  |  |  |  |  |  |
| *Acinetobacter baumannii* NCCP 12277 | 1.5 | 0.5 | 0.125 | 0.33 | 0.455 | + |
| *Acinetobacter baumannii* NCCP 14606 | 2 | 0.5 | 0.125 | 0.25 | 0.375 | + |
| *Acinetobacter baumannii* NCCP 14654 | 2 | 0.5 | 0.25 | 0.25 | 0.5 | + |
| *Acinetobacter baumannii* NCCP 14782 | 2 | 0.5 | 0.125 | 0.25 | 0.375 | + |
| *Acinetobacter baumannii* NCCP 15990 | 1.5 | 0.5 | 0.125 | 0.33 | 0.455 | + |
| *Acinetobacter baumannii* NCCP 15998 | 2 | 0.5 | 0.125 | 0.25 | 0.375 | + |
| *Acinetobacter baumannii* F-1208 | 4 | 1 | 0.25 | 0.25 | 0.5 | + |
| *Acinetobacter baumannii* F-1379 | 2 | 0.5 | 0.125 | 0.25 | 0.375 | + |
| *Acinetobacter baumannii* F-1410 | 2 | 0.5 | 0.125 | 0.25 | 0.375 | + |
| *Acinetobacter baumannii* NCCP 14608 | 2 | 2 | 0.125 | 1 | 1.125 | − |
| *Acinetobacter baumannii* NCCP 14655 | 2 | 2 | 0.125 | 1 | 1.125 | − |
| *Acinetobacter baumannii* NCCP 15987 | 2 | 2 | 0.25 | 1 | 1.25 | − |
| *Acinetobacter baumannii* NCCP 16006 | 2 | 2 | 0.25 | 1 | 1.25 | − |
| *Acinetobacter baumannii* NCCP 16010 | 2 | 2 | 0.25 | 1 | 1.25 | − |
| *Acinetobacter baumannii* NCCP 16011 | 2 | 2 | 0.25 | 1 | 1.25 | − |

**Supplementary Table S5:** Fractional inhibitory concentration (FIC) of PMB and 6-bromo-2-naphthol from *S. armeria* extract. FIC^a^, The concentration of 6-bromo-2-naphthol and 1-naphthol used for FIC was fixed at 32 μg/mL and 64 μg/mL, respectively. FIC^b^, The minimum inhibitory concentration (MIC) of polymyxin B was not determined. FIC index, calculated as the sum of FICs of 6-bromo-2-naphthol (or 1-naphthol) and polymyxin B.

| **Agent (polymyxin B)** | **MIC** | | **FIC^a^** | **FIC^b^** | **FICI** | **Result** |
| --- | --- | --- | --- | --- | --- | --- |
|  | **Alone** | **Combination** |  |  |  |  |
| 6-Bromo-2-naphthol | 64 | 16 | 0.25 | 0.25 | 0.5 | Synergy |
| 1-Naphthol | 128 | 32 | 0.25 | 0.25 | 0.5 | Synergy |
